# Verarbeitungseigenschaften und Viskositäten von PMMA-Knochenzementen

**DOI:** 10.1007/s00132-023-04450-x

**Published:** 2023-10-30

**Authors:** Christian Paul, Erwin Steinhauser, Klaus-Dieter Kühn

**Affiliations:** 1 Heraeus Medical GmbH, Wehrheim, Deutschland; 2 Fakultät für angewandte Naturwissenschaften und Mechatronik, Hochschule München, München, Deutschland; 3 Universitätsklinik für Orthopädie und Traumatologie, Medizinische Universität Graz, Graz, Österreich

**Keywords:** Einflussfaktoren, Polymethylmethacrylat, Klebephase, Verarbeitungsphase, Aushärtezeit, Influencing factors, Polymethylmethacrylate, Doughing time; Sticky phase, Working phase, Setting time

## Abstract

Verarbeitungseigenschaften von PMMA-Knochenzementen können in vier Phasen unterteilt werden: 1. Anmischung, 2. Wartephase, 3. Verarbeitungsphase sowie 4. Aushärtephase. Jede dieser Phasen unterliegt einer Reihe von äußeren Einflussfaktoren, beispielsweise Temperatur und Feuchte, die im Rahmen der Anwendung zu beachten sind. Gebrauchsanweisungen der Hersteller von PMMA-Knochenzementen sowie von Misch- und Applikationssystemen enthalten wichtige Hinweise zur korrekten Anwendung. Für den sicheren Ablauf im Operationssaal sind die Verarbeitungseigenschaften der PMMA-Knochenzemente und mögliche Einflussfaktoren auf den Aushärtungsverlauf von großer Bedeutung. Kenntnisse zu Viskosität und Konsistenz des PMMA-Knochenzementes von der Teigphase bis zur vollständigen Aushärtung erleichtern das Vorbereiten und Applizieren, was langfristig die an PMMA-Zemente gestellten Anforderungen im Hinblick auf Funktion und Standzeit des Implantats signifikant verbessert.

## Abstract (en)

Processing properties of PMMA bone cements can be divided into four phases: 1. mixing, 2. waiting, 3. processing and 4. curing. Each of these phases is subject to several external influencing factors, such as temperature and humidity, which must be considered during application. Instructions for use provided by the manufacturers of PMMA bone cements and mixing and application systems contain important information on correct application. The processing properties of PMMA bone cements and possible factors influencing the curing process are of great importance for safe procedures in the operating room. Knowledge of the viscosity and consistency of the PMMA bone cement from the dough phase to complete curing facilitates preparation and application, which in the long term significantly improves the requirements placed on PMMA cements regarding the function and service life of the implant.

Knochenzemente aus PMMA lassen sich nach der Anmischung der beiden Komponenten (Pulver + Flüssigkeit) wie eine Knetmasse modellieren und bieten nach Implantation die Möglichkeit, die Prothese bestmöglich zu verankern. Die Verarbeitungseigenschaften von PMMA-Knochenzement sind jedoch zeitlich begrenzt. Anwender im Operationssaal sollten den Verlauf von der Teigphase bis zur Aushärtung kennen und einschätzen, wann das Implantat im Zement in seiner finalen Position fixiert ist. Dieser Beitrag soll zeigen, welche Faktoren die Verarbeitungseigenschaften beeinflussen.

## Einleitung

Knochenzemente aus PMMA sind Zwei-Komponenten-Kunststoffe und werden den Chirurgen als Pulverbeutel (Polymer) und Flüssigkeitsampulle (Monomer) zur Verfügung gestellt [4, 17]. Nach dem Mischen und Anquellen der Pulver in der Flüssigkeit entsteht zunächst ein Zementteig mit niedriger Viskosität, die ständig und unaufhaltsam ansteigt, bis der Teig vollständig aushärtet [4, 18, 19]. Die Verarbeitungseigenschaften von PMMA-Knochenzementen sind abhängig vom Viskositätsverlauf, der wiederum wesentlich von der polymeren Zusammensetzung des jeweiligen PMMA-Zementes bestimmt wird. Der Hauptbestandteil aller PMMA-Knochenzemente ist Polymethacrylsäuremethylester (PMMA), industriell bekannt als Plexiglas® [17]. Bereits in den 1950er-Jahren kamen Wissenschaftler zu der Erkenntnis, dass sich aus Polymethacrylsäuremethylester (PMMA) und seinen Grundbausteinen, dem flüssigen Methacrylsäuremethylester (MMA) nach Zugabe spezieller Wirk- [20] und Hilfsstoffe ein Teig erzeugen lässt, der alle Voraussetzungen erfüllt, um metallische Implantate im Knochen zu verankern [18]. Hinter diesem Erfolgskonzept verbergen sich im Wesentlichen die Verarbeitungseigenschaften der PMMA-Zemente [4]. Damit PMMA-Zemente im Operationssaal sicher angewendet werden, sollten sie im Rahmen eines bestimmten Zeitfensters angemischt und verwendet werden, bevor der Zementteig zu hochviskos ist und der Zement vollständig aushärtet.

Für das Operationspersonal sowie Chirurginnen und Chirurgen ist es entscheidend zu wissen, in welchem Verarbeitungszeitraum (1. Anmischung, 2. Wartephase, 3. Verarbeitungsphase und 4. Aushärtephase) sich der vorliegende Knochenzement befindet und welche Faktoren diesen Zeitraum beeinflussen können [18]. Im Rahmen dieses Beitrags werden die Effekte und Einflussfaktoren auf Verarbeitungseigenschaften von PMMA-Knochenzementen vorgestellt.

### Anmischvorgang

Beim Anmischen der beiden Komponenten der PMMA-Knochenzemente werden die Polymerperlen der Pulverkomponente an der Oberfläche durch das flüssige MMA angelöst. Hierbei dringt das MMA nur wenige Mikrometer in die PMMA-Perlen ein, wodurch diese leicht anquellen. Durch diesen physikalischen Prozess erhöht sich allmählich die Viskosität des Pulver-Flüssigkeit-Gemischs, wodurch eine knet- und verarbeitbare Masse entsteht [21]. Die Geschwindigkeit des Anquellprozesses ist allein von der Zusammensetzung und Beschaffenheit der Polymerperlen sowie deren Co-Polymeren abhängig. Diese variieren bei PMMA-Knochenzementen von Produkt zu Produkt erheblich [17]. Darüber hinaus spielt auch die Größe und damit der Herstellprozess der eingesetzten Polymerperlen eine eminent wichtige Rolle. Größere PMMA-Kugeln benötigen für diesen Anquellprozess mehr Zeit. Auf diese Weise können beispielweise niedrigviskose PMMA-Zemente (LV-Zemente) hergestellt werden, die sich durch eine flüssige bis niedrigviskose Anquellphase charakterisieren lassen [4]. Durch das Hinzufügen von PMMA-Perlen eines geringeren Durchmessers beschleunigt sich der Anquellvorgang und die Viskosität steigt an. Zemente dieser Variante werden auch als hochviskose Knochenzemente (HV-Zemente) bezeichnet. Durch eine festgelegte Rezeptur sowie validierte Herstellungsverfahren können die Hersteller von Knochenzementen sicherstellen, dass die Zemente eine gleichbleibende Viskosität verbunden mit reproduzierten Verarbeitungseigenschaft aufweisen.

Je nach Art des Anmischgefäßes und dem Einsatz von Vakuum während der Anmischung, kann es zu Unterschieden im Verarbeitungs- und Aushärteverhalten kommen [21, 32]. Sigmund et al. [32] zeigten, dass sowohl das Anmischen unter Vakuum als auch die Mischbewegungen einen signifikanten Einfluss auf die Verarbeitungseigenschaften haben können. Es empfiehlt sich daher, die vom Hersteller vorgegebene Mischfrequenz beim Einsatz von Mischsystemen einzuhalten. Eine zu starke und schnelle Mischgeschwindigkeit kann durch die höhere eingebrachte Energie den Aushärtevorgang beschleunigen, während ein zu langsames Mischen mit niedrigem Energieeintrag zu einem inhomogenen Teig und insbesondere zu einer veränderten Klebephase führen kann [32].

### Polymerisation

Während des Anquellprozesses des Polymers im Monomer startet bereits der Polymerisationsvorgang von flüssigen MMA zu festem PMMA, der die Knochenzemente zur Aushärtung bringt. Diese Polymerisation hängt vom eingesetzten Initiatorsystem in der Pulver- und Flüssigkeitskomponente ab. Alle im Markt befindlichen Knochenzemente basieren auf dem gleichen Initiatorsystem bestehend aus Dibenzoylperoxid (DBPO), welches im Rahmen des Herstellungsprozesses der Pulverkomponente zugemischt wird. Neben dem Initiator besteht das System noch aus einem Aktivator als Starter des Polymerisationsvorgangs. Bei dem Aktivator handelt es sich üblicherweise um N,N-Dimethyl-p-toluidin (DmpT), das dem MMA der flüssigen Komponente zugegeben wird [17]. Sobald die beiden Komponenten beim Anmischvorgang in Kontakt kommen, reagieren das DBPO und das DmpT miteinander und der Polymerisationsvorgang wird gestartet. Je nach Zusammensetzung der Zemente kann das Initiatorsystem komplett abreagieren [4, 7, 18, 21]. PMMA-Knochenzemente unterscheiden sich im DBPO-DmpT-Verhältnis. So weisen mache Zemente einen Überschuss an DBPO auf, andere einen an DmpT [34].

## Viskositäten von Knochenzementen

Knochenzemente aus PMMA werden üblicherweise in die drei Viskositätskategorien niedrig-, mittel- und hochviskose Varianten eingeteilt [18]. Je nach Einsatzgebiet obliegt es dem Anwender, sich für eine Variante zu entscheiden. Hochviskose Knochenzemente werden weltweit in den meisten orthopädischen Verankerungen von Endoprothesen eingesetzt [18, 22]. Sie werden mit und ohne Wirkstoffe angeboten [21]. HV-Zemente erreichen in kurzer Zeit das Ende ihre Klebephase und sind daher schnell einsatzbereit. Zudem zeichnen sie sich durch eine lange Verarbeitungsphase aus, die den Chirurgen Zeit geben, die Prothesen sicher im Knochen zu fixieren, bevor der Zement aushärtet.

Zur Verbesserung der Zementqualität wurden in den 1980er- bis 1990er-Jahren, im Rahmen der modernen Zementiertechnik erstmals Kartuschenmischsysteme für PMMA-Knochenzemente entwickelt [21]. Der Einsatz von zwei bis drei Packungen hochviskoser PMMA-Knochenzemente in einer Anmischkartusche erwies sich oftmals als schwer und ging mit einem erhöhten Kraftaufwand für das Anmischen einer homogenen Teigmischung einher. Neben einer Kühlung der Zementkomponenten wurden mittelviskose Varianten entwickelt, die auch ohne Kühlung in den Anmischsystemen verwendet werden konnten [24]. Durch eine niedrigere Anfangsviskosität ermöglichte diese Art von Knochenzement (MV-Variante) eine leichtere Anmischung und Extrusion des Zementteigs [18]. Heutzutage werden MV-Knochenzemente insbesondere in der Knieendoprothetik eingesetzt. Durch die mittlere Viskosität können diese Zemente in einer frühen, noch niedrigviskosen Phase, zunächst auf das Metallimplantat aufgebracht und anschließend bei etwas höherer Viskosität, nach dem Ende der Klebephase, implantiert werden. Die beiden mit Zement benetzten Kontaktflächen (Implantat-Knochen) werden anschließend zusammengebracht und ermöglichen eine bestmögliche Benetzung und Verzahnung im Knochen [19, 21, 22].

**Abb. 1:**
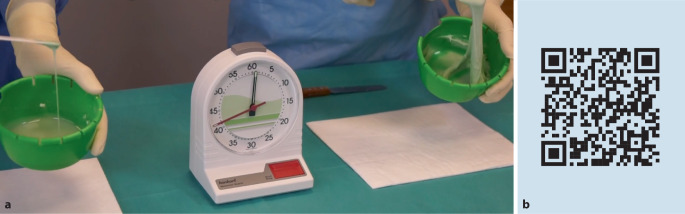
**a** Videostill zu Trainingsvideo 1 „Viskositäten“. **b** QR-Code zu Trainingsvideo 1 „Viskositäten“: https://heraeus.wistia.com/medias/kp2okzctpr. (**a**, **b** mit freundl. Genehmigung, © Heraeus Medical GmbH, alle Rechte vorbehalten)

Niedrigviskose Zemente (LV) werden in der Regel dann eingesetzt, wenn es sich um kleine Kavitäten, wie Ellbogen oder Fingergelenke, handelt (Abb. 1). Die niedrige Viskosität ermöglicht es so, den Knochenzement auch durch dünne Schnorchel oder Kanülen zu applizieren [18]. In der Wirbelsäulenchirurgie werden niedrigviskose Varianten mit langanhaltender flüssiger Viskositätsphase einsetzt, um erkrankte und zerstörte Wirbelkörper aufzurichten und zu stabilisieren [18, 22, 32]. In Tab. 1 werden die verschiedenen Viskositäten sowie Eigenschaften und Beispiele aufgeführt.

**Tab. 1:** Übersicht Viskositäten von Knochenzementen [18]

Viskosität	Ende Klebephase bei RT (min)	Verhalten	Beispiel
Niedrig	3,0–4,0	Lange flüssige Phase In Verarbeitungsphase starker Viskositätsanstieg Schnelle Aushärtungsphase	Palacos LV/LV+G, CMW 3, Osteopal V, Tianjin Joint Cement
Mittel	1,5–3,0	Niedrige Anfangsviskosität In Verarbeitungsphase gleichmäßig moderater Viskositätsanstieg	Palacos MV/MV+G, Refobacin Bone Cement R, Simplex P
Hoch	0,5–1,5	Kurze Benetzungsphase und damit eine kurze Klebephase Längere Verarbeitungsphase In Verarbeitungsphase gleichmäßig moderater Viskositätsanstieg	Palacos R/R+G, Copal G+C/G+V, SmartSet GHV

Angaben zu Verarbeitungseigenschaften von PMMA-Knochenzementen werden stets unter standardisierten Laborbedingungen (in der Regel 23 °C und 55 % relativer Feuchte) erhoben und sollten als freigaberelevante Parameter für Produkte gelten [15, 16]. Der Aushärtungsverlauf der Knochenzemente ist in vivo insbesondere von den klimatisierten Operationsbedingungen und vom Handling der Anwender abhängig. Umgebungs- und Lagerbedingungen sollten bereits vor der Verwendung der Knochenzemente im Operationssaal beachtet werden.

Grundlage für die Einteilung der in Tab. 1 aufgeführten Viskositätskategorien ist die Bestimmung der Verarbeitungseigenschaften der Knochenzemente. Der Polymerisationsprozess der PMMA-Knochenzemente ist in vier Phasen unterteilt, welche in Tab. 2 aufgeführt sind [18].

**Tab. 2:** Schematische Darstellung der Polymerisationsvorgangs

Phase	Name	Moleküle	Beschreibung
I	Anmischphase	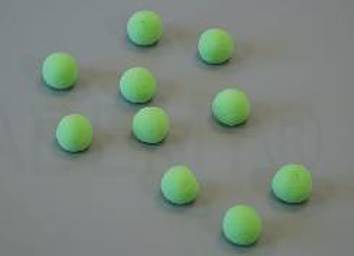	Benetzung der Polymere mit dem Monomer Zementgemisch ist flüssig, hat niedrigste Viskosität Start der Polymerisation Zementteig noch nicht klebefrei
II	Klebephase (Wartephase)	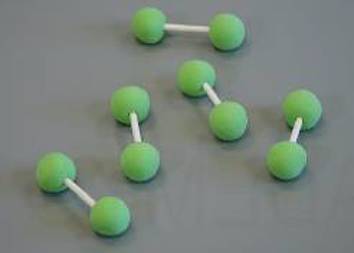	Oberflächliches Anquellen der PMMA-Perlen Durch Polymerisation entstehen erste Ketten Anstieg der Viskosität Teig bis zum Ende der Phase noch nicht klebefrei
III	Verarbeitungs- und Applikationsphase	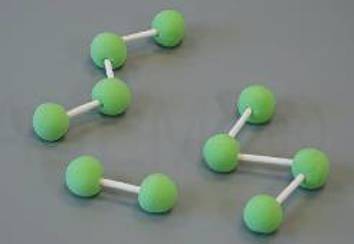	Zementteig kann in vivo eingesetzt werden Polymerketten werden länger Reduzierte Mobilität der Moleküle Viskositätsanstieg im Teig Wärmeentwicklung durch exotherme Reaktion
IV	Aushärtephase	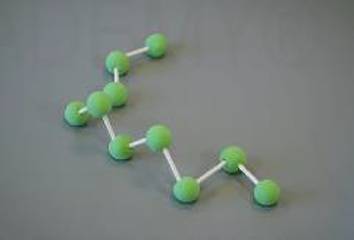	Kettenwachstum ist abgeschlossen Keine Molekülbewegungen mehr möglich Temperaturmaximum wird erreicht Zement ist ausgehärtet

*PMMA* Polymethacrylsäuremethylester

### Bestimmung der Verarbeitungsphasen

**Abb. 2:**
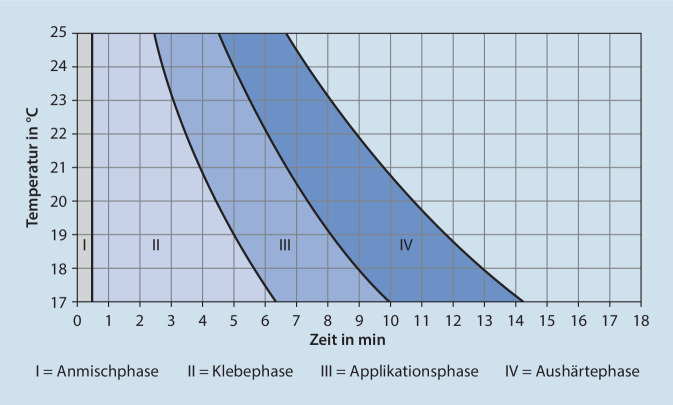
Typische Abbildung der Verarbeitungseigenschaften eines Knochenzementes [18]

Alle PMMA-Zemente im Markt durchlaufen die hier aufgeführten Polymerisationsphasen. Trotz gleicher Viskositätsklassifizierung (HV, MV, LV) unterscheiden sich handelsübliche Knochenzemente je nach Zusammensetzung im Hinblick auf die in Tab. 2 gezeigten Phasen. Um dem Anwender eine Orientierungshilfe zu geben, empfiehlt die ISO 5833 für Knochenzemente [15] eine detaillierte, möglichst grafische Darstellung der Handhabungseigenschaften in Abhängigkeit von der herrschenden Umgebungstemperatur in der Gebrauchsanweisung abzubilden (Abb. 2).

Umgebungs- und Lagerbedingungen haben großen Einfluss auf die Verarbeitungseigenschaften

Um den Verarbeitungszustand der Knochenzemente reproduzierbar bestimmen und einschätzen zu können, wird nach dem Zusammenbringen der Pulver- und Flüssigkeitskomponente die Zeitmessung gestartet. Für das geschulte Operationspersonal sind Phase I und II entscheidend [18]. Am Ende der Phase II liegt der klebefreie Zementteig vor und kann den Chirurginnen und Chirurgen für die Implantation zur Verfügung gestellt werden. Diese orientieren sich dann im Wesentlichen an der Verarbeitungs- und Applikationsphase (Phase III). Sie gibt an, wie lange der Zement modelliert und eingebracht werden kann. Für den sicheren Erfolg der Operation ist es essenziell, dass der Zementteig optimal im spongiösen Knochen eingedrungen ist (2–5 mm). Am Ende der Phase III sollte sich das Implantat in der finalen Position befinden und unter keinen Umständen mehr bewegt werden [2]. Nachteilig wirkt sich ein zu frühes Einbringen des Zementteigs aus. Dies kann zum Wegfließen des Zements führen. Zudem kann der noch zu niedrigviskose Teig dem Blutungsdruck nicht standhalten und sich mit Blut vermischen. Bei einer zu späten Verwendung eines zu hochviskosen Teigs kann es zu einer schwachen Interdigitation kommen und der Verbund zwischen Zement und Knochen beziehungsweise Zement und Implantat ist unzureichend [19, 22].

Die ISO 5833 [15] bietet eine praxisbezogene und einfache Standardmethode, um das Ende der Klebephase („doughing time“) zu ermitteln (bekannt auch als Doctor’s-Fingertest). Methoden, mit denen man alle vier Phasen (Tab. 2) bestimmen kann, werden in internationalen Normen nicht beschrieben. In der Literatur gibt es jedoch ein allgemeines Vorgehen [18], welches sich für die genaue Ermittlung der Verarbeitungszeiten anbietet. Interessant sind hierbei die verschiedenen Einflussfaktoren, insbesondere im Hinblick auf die Umgebungsbedingungen, da nicht jeder Operationssaal klimatisiert ist und über die gesamte Operationsdauer gleichbleibende Bedingungen aufweist. In den folgenden Kapiteln werden die Bestimmung der oben genannten vier Phasen (Tab. 2) sowie die relevanten Einflussfaktoren beschrieben [18, 21].

### Anmischphase

Bereits innerhalb der Anmischphase (Phase I) gibt es bei den PMMA-Knochenzementen verschiedener Hersteller enorme Unterschiede. Einige Zemente lassen sich leicht mischen und homogenisieren (Palacos®, Heraeus Medical, Wehrheim, Deutschland, SmartSet®, DePuy, Raynham, MA, USA), andere besitzen eine voluminöse Pulverkomponente und sind anfangs eher trocken (Simplex®, Stryker, Duisburg, Deutschland, Cemex®). Eine mögliche Erklärung für dieses trockene und voluminöse Anquellverhalten könnte die Verwendung von Styrol-Copolymeren bei Simplex und Cemex-Zementen sein. Solche Angaben zur Zusammensetzung der Polymere sind aber durch das Medizinproduktegesetz nicht mehr vorgeschrieben.

In der Regel wird die Flüssigkeit vorgegeben und das Pulver zudosiert. Nur wenige Ausnahmen schreiben die umgekehrte Reihenfolge vor (z. B. Simplex®).

Gemäß einer Umfrage zur Zementiertechnik in der Hüftendoprothetik in Deutschland [5] wurde festgestellt, dass nur zwei Drittel der Anwender die vom Hersteller vorgegeben Mischzeiten einhalten. Das Nichteinhalten dieser Vorgaben kann jedoch einen erheblichen Einfluss auf die späteren mechanischen Eigenschaften des ausgehärteten Knochenzementes haben [23]. Bei zu langem oder „zu gründlichem“ Mischen können kleine Luftblasen in den Teig eingebracht werden, was im Resultat zu einer geringeren Dichte und erhöhter Porosität führt [6, 8, 27, 35].

Neben der Art und Weise des Anmischens kann zudem auch die Geometrie des Anmischgefäßes, des eingesetzten Spatels sowie besonders die Mischgeschwindigkeit einen Einfluss auf den ausgehärteten Zement haben [10, 32]. Aus diesen Gründen ist es wichtig, dass sich der Anwender vor dem Einsatz der Zemente auch mit der Gebrauchsanweisung und dem jeweiligen Mischsystem vertraut macht. Studien des sogenannten Schwedenregisters haben gezeigt, dass zunehmende Erfahrung im Umgang mit Knochenzementen und Mischsystemen auch zu besseren klinischen Langzeitergebnissen führt [30].

### Klebephase

**Abb. 3:**
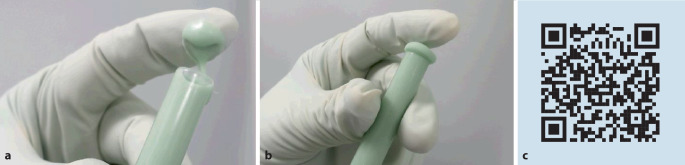
**a**, **b** Bestimmung der Klebephase mittels Doctor’s-Fingertest. **c** QR-Code zu Trainingsvideo 2 „Klebephase“: https://heraeus.wistia.com/medias/votxumxaii (**a–c** mit freundl. Genehmigung, © Heraeus Medical GmbH, alle Rechte vorbehalten)

Je nach Zementzusammensetzung benötigt das MMA nach Mischbeginn unterschiedlich viel Zeit, um die Polymerperlen oberflächlich anzulösen und erste Polymerketten auszubilden. Dieser Anquellvorgang ist insbesondere von der Korngrößenverteilung der Polymerpulver, der molaren Masse und dem Aktivator-Initiator-Verhältnis abhängig [34]. Solange noch viele freie MMA-Moleküle in dem flüssigen bis niedrigviskosen Teig vorliegen, klebt der Knochenzement oberflächlich noch zu stark und sollte in diesem Zustand nicht verarbeitet oder in den Knochen eingebracht werden. In dieser frühen Phase tritt ein visuell gut erkennbarer glänzender Effekt an der Oberfläche des Zementes auf. Nach kurzer Zeit wird der Zementteig matt und oberflächlich klebefrei. Die oberflächliche Klebfreiheit ist definitionsgemäß die ISO „doughing time“ [19, 21]. Der Zustand der Klebfreiheit wird in der Praxis leider oft nicht korrekt bestimmt. Solche Fehlinterpretationen führen leicht dazu, dass der Teig zu früh eingebracht wird. Die Wartephase soll als Sicherheitspuffer dazu dienen, einen Zementteig nicht „zu flüssig“ zu verwenden. Wissenswert für die Beurteilung der Klebfreiheit sind physikalische Eigenschaften des MMA. Beim MMA handelt es sich um ein flüchtiges Lösemittel mit vergleichsweise hohem Dampfdruck [3]. Auf vergrößerten Oberflächen an den Teigschichten kommt es daher rasch zu einem Verdunstungseffekt. Um das Ende der Klebephase richtig zu bestimmen, schreibt nicht zuletzt auch die ISO 5833 [15] vor, stets eine frische Zementoberfläche zu untersuchen und mit einer unbenutzten Fläche des Handschuhes den Test durchzuführen. Dieser Test wird in der Literatur [18, 21] auch in Anlehnung an die im Operationssaal durchgeführte Praxis als „Doctor’s-Fingertest“ beschrieben. Unabhängig von dem eingesetzten Anmischgefäß oder verwendeten Applikationssystem sollte für jede Bestimmung der Klebfreiheit eine „frische“ Zementschicht herangezogen werden. Der Zement sollte, beispielsweise bei der Anmischung in einem System wie in Abb. 3 gezeigt, ca. 1 cm aus dem Schnorchel extrudiert werden und anschließend auf Klebfreiheit untersucht werden. Ist der Zeitpunkt noch nicht erreicht, wird der noch klebrige Zement verworfen und erneut 1 cm nachextrudiert. Dieser Vorgang wird so lange wiederholt, bis das Ende der Klebephase erreicht ist. Zwischen den einzelnen Testungen sollten gemäß ISO-Norm ca. 15 s liegen. Das Ende der Klebephase markiert zudem auch das Ende der Wartephase, ab der der Zement eingesetzt wird.

Für eine sichere Anwendung ist es unerlässlich, vor dem Einbringen das Ende der Klebephase abzuwarten. Andernfalls kann der Knochenzement durch eine zu geringe Anfangsviskosität dem Blutungsdruck im Knochen nicht standhalten. Es kann zu kleinen Bluteinschlüssen in der Zementmatrix kommen, welche als Schwachstellen einen negativen Einfluss auf die mechanische Langzeitstabilität des Implantats haben können [28, 33]. Die Bestimmung der Klebephase nach ISO 5833 [15] sagt demnach nichts über den Teigzustand im Rahmen der Verarbeitungsphase aus, sondern lediglich über die früheste Einsatzbereitschaft des Zementes.

### Verarbeitungs- und Applikationsphase

Nach dem Ende der Klebephase kann der Knochenzement unter geringem Druck modelliert und beispielsweise in der Knieendoprothetik auf die Unterseite des metallischen Tibiaplateaus oder in den Knochen eingebracht werden. Es hat sich zudem gezeigt, dass langsames Kneten nach der Klebephase die Porosität weiter reduzieren kann [10]. Wie in Tab. 1 gezeigt, besitzen Knochenzemente je nach Viskosität nach der Klebephase unterschiedlich lange Verarbeitungsphasen. Neben Lager- und Umgebungsparametern kann auch das eingesetzte Mischsystem einen Einfluss auf die Verarbeitungszeiten haben [32, 35]. Derzeit gibt es noch keine bekannte Methode, mit der sich die unterschiedlichen und für die Praxis entscheidenden Parameter reproduzierbar abbilden lassen. Es ist daher sinnvoll und empfehlenswert, sich in praktischen Schulungen die Verarbeitungseigenschaften von PMMA-Knochenzementen anzueignen.

**Abb. 4:**
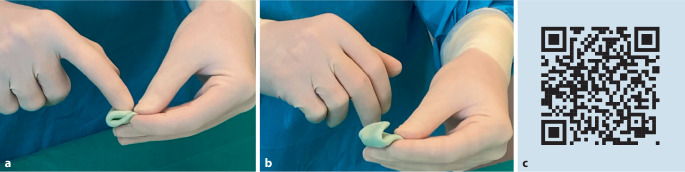
**a**, **b** Bestimmung des Verarbeitungsendes durch den „Cookie-Test“. **c** QR-Code zu Trainingsvideo 3 „Verarbeitungsphase“: https://heraeus.wistia.com/medias/helrl3pwbd. (**a**–**c** mit freundl. Genehmigung, © Heraeus Medical GmbH, alle Rechte vorbehalten.)

Gegen Ende der Verarbeitungsphase ist die Viskosität sehr hoch, weil die vielen PMMA-Ketten durch die voranschreitende Polymerisation immer länger und dadurch unbeweglicher geworden sind. Infolgedessen stellt sich ab einem bestimmten Viskositätszeitpunkt eine gummiartige Rückstellkraft des Materials ein. Um das Ende der Verarbeitungsphase korrekt bestimmen zu können, bietet es sich an, aus einer kleinen Zementprobe eine flache Scheibe in Form eines „Cookie“ zu formen. Für die Bestimmung faltet man wie in Abb. 4 gezeigt die Scheibe unter leichtem Druck zusammen.

Die Bestimmung des Verarbeitungsendes durch den Anwender ist stets subjektiv

Solange die beiden Enden der Zementscheibe noch aneinanderhaften, kann das Material noch verarbeitet und die Prothese positioniert werden. Sobald sich die Knochenzementprobe nicht mehr faltenfrei vereinigen lässt und durch die Rückstellkraft wieder „aufspringt“, ist das Ende der Verarbeitungsphase erreicht. Ab diesem Punkt sollte das Implantat nicht mehr bewegt werden, da es sonst zu einer Lockerung der Prothese kommen kann [2, 18].

**Abb. 5:**
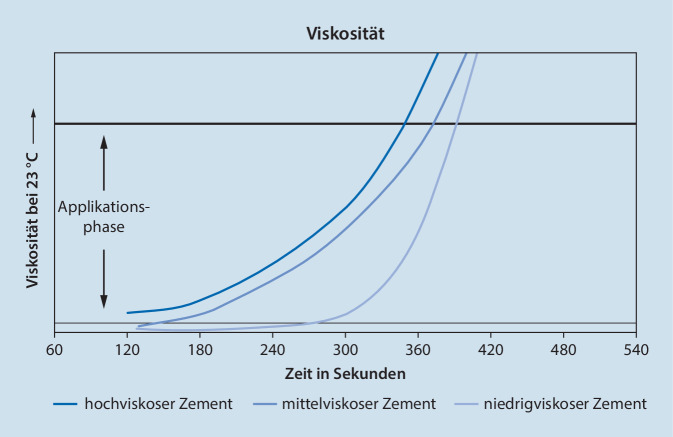
Viskositätsprofil von hoch-, mittel- und niedrigviskosem Zement, geprüft mit Gelnorm-Med® (Gel Instrumente, Oberuzwil, Schweiz), Stempel 6 cm^2^, Geschwindigkeit 0,03 mm/s, relative Feuchte 55 ± 5 %, Viskosität gemessen als Gegendruckkraft in N [26]

Da die Bestimmung des Verarbeitungsendes durch den Anwender stets subjektiv erfolgt, kann es hier je nach Erfahrung und Behandlung der Knochenzementprobe zu Unterschieden kommen. Eine genormte Methode für die maschinelle Bestimmung der Verarbeitungseigenschaften der Knochenzemente gibt es derzeit noch nicht [18]. Dennoch eignen sich für die Ermittlung der Viskosität handelsübliche Laborgeräte zur Kraftmessung von pastösen Materialen. Durch die aushärtenden Eigenschaften der Zemente sollten hier aber in erster Linie Einweggefäße und Messwerkzeuge zum Einsatz kommen. Durch diese maschinelle Bestimmung der Verarbeitungseigenschaften lassen sich Fehlerquellen – insbesondere der „Faktor Mensch“ – weitestgehend ausklammern. In Abb. 5 werden drei unterschiedlichen Viskositätsverläufe handelsüblicher Knochenzemente dargestellt.

Viskositätsmessungen von dynamischen Systemen, also sich permanent veränderten Viskositäten, sind technisch eine große Herausforderung. Solche Viskositätsuntersuchungen dürfen daher zwingend nur unter standardisierten Laborbedingungen durchgeführt werden. Idealerweise werden Standardpackungen (40 g Pulver, 20 ml Flüssigkeit) verwendet, da bereits geringe Abweichungen des Pulver-Flüssigkeits-Verhältnisses zu signifikanten Abweichungen führen können. Zudem ist bei relativ großen Zementmassen unter In-vitro-Tests (40 g Pulver, 20 ml Flüssigkeit) mit mehr exothermer Energie zu rechnen, was die Reaktionskinetik wiederum beschleunigt [18]. Darüber hinaus sind die Flächenverhältnisse von Gefäß und Messwerk nicht gleich der Kontaktflächen zu Knochen und Prothese. Auch findet diese Testung an der Luft und nicht ein einem feuchten Milieu statt [26].

Daher ist es ratsam, die Zementprobe unter Operationsbedingungen nicht konstant in der geschlossenen Hand (Wärmezufuhr bewirkt Beschleunigung) oder auf einem kalten Operationstisch (führt zu einer Verlangsamung) abzulegen. Während der Aushärtung empfiehlt sich ein Mittelweg durch Ablage auf einem Tuch und gelegentlichem Testen und Falten mit dem Finger. Dennoch ist auch dieser Mittelweg nur als eine Annäherung an die eigentlichen In-vivo-Bedingungen anzusehen, die je nach Art der Operation, von der eingesetzten Größe des Metallimplantats als Wärmeleiter sowie der Schichtdicke des Knochenzementes abhängig sind. Auch bei der Bestimmung des Endes der Verarbeitungsbreite ist eine intensive Schulung empfehlenswert, die einen sicheren Umgang mit dem PMMA-Zement erleichtert.

### Aushärtungsphase

**Abb. 6:**
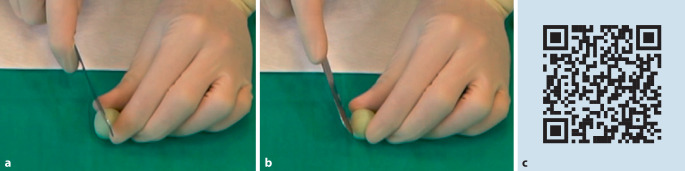
**a**, **b** Bestimmung des Aushärtungszustandes durch Einritzen im Operationssaal. **c** QR-Code zu Trainingsvideo 4 „Aushärtephase“: https://heraeus.wistia.com/medias/kdswo76ty5. (**a**–**c** mit freundl. Genehmigung, © Heraeus Medical GmbH, alle Rechte vorbehalten.)

Die Aushärtungsphase beschreibt den Zustand, bei dem der Knochenzement vollständig ausgehärtet ist [18]. Um eine grobe Einschätzung über den Zeitpunkt der vollständigen Aushärtungszeit (ISO „setting time“) zu erhalten, weisen die Hersteller von Knochenzementen diesen Zeitpunkt bei verschiedenen Temperaturen in den entsprechenden Gebrauchsanweisungen aus. Hierzu muss angemerkt werden, dass diese Daten, wie auch bei den restlichen Phasen der Verarbeitung, für standardisierte Laborbedingungen (23 °C ± 1 °C und > 40 % relativer Feuchte) gelten. Die Bestimmung dieses Aushärtungszeitpunktes im Labor ist nach ISO 5833 mit einer vollständigen Packung Knochenzement durchzuführen [15]. Im Rahmen einer Operation verbleibt oft nur eine kleine Zementprobe (Dicke ca. 2–5 mm), um die Bestimmung der vollständigen Aushärtung durchzuführen. Die Bestimmung des Zeitpunktes kann an beiden In-vitro-Proben durch Einritzen mit einer Schere oder einem Spatel erfolgen. Entsteht keine Kerbe, ist der Zement in vitro vollständig ausgehärtet (Abb. 6). Alternativ kann auch ein Klangtest erfolgen, bei dem die ausgehärtete Zementprobe leicht auf den Metalltisch geklopft wird. Ist ein typischer „keramischer Klang“ zu hören, ist das Material vollständig ausgehärtet [21].

**Abb. 7:**
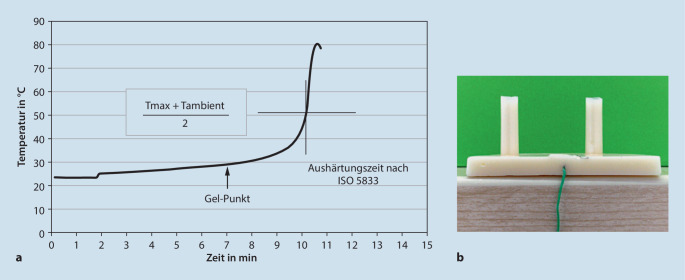
Typische ISO-Temperatur- und Aushärtungskurve (**a**) eines Zementprüfkörpers (**b**)

Auch in den gängigen ISO/ASTM-Normen für PMMA-Knochenzemente [1, 15] gibt es einen Test zur Ermittlung der Aushärtezeit („setting time“). Hierbei wird eine definierte Schichtdicke des Zementes in einer Teflonform zur Aushärtung gebracht. Während der Aushärtung wird der Temperaturanstieg durch einen Temperaturfühler im Inneren der Zementprobe aufgezeichnet (Abb. 7).

Eine genaue In-vivo-Bestimmung der „setting time“ ist ggf. am sichtbaren Zementmantel am Patienten möglich. Es ist im Operationssaal ratsam abzuwarten, bis das Restmaterial am Tisch vollständig ausgehärtet ist. Wurde das Restmaterial ohne zusätzlichen Energieeintrag (Kneten, in der Hand halten usw.) behandelt, spricht die höhere Körpertemperatur dafür, dass der Zement im Körper bei Erreichen der Aushärtungszeit („setting time“) des Restmaterials ebenfalls ausgehärtet ist.

Die Bestimmung aller Verarbeitungsphasen unterliegt einigen Einflussfaktoren, auf die im nachfolgenden Kapitel eingegangen wird.

## Anwendung im Operationssaal

Wie bereits erwähnt, gibt es bei der Ermittlung der Verarbeitungseigenschaften von Knochenzementen enorme Unterschiede zwischen der Bestimmung im Operationssaal und unter standardisierten Laborbedingungen. Generell unterliegen Operationsproben aufgrund ihrer geringen Masse größeren Schwankungen durch relevante Einflussfaktoren [18].

### Einflussfaktoren

Es gibt eine Reihe von Faktoren, die die Verarbeitungseigenschaften der Knochenzemente unter In-vivo-Bedingungen beeinflussen können (Tab. 3).

**Tab. 3:** Übersicht Einflussfaktoren und Auswirkung auf Verarbeitungseigenschaften [18]

Einflussfaktor	Auswirkung auf Verarbeitungseigenschaft
Temperatur (Umgebung und Material)	Aushärtung (ISO) bei 23 °C: z. B. nach 6–7 min Aushärtung (ISO) bei 2–6 °C z. B. nach 12–14 min
Vorgewärmtes oder gekühltes Mischgefäß bzw. Applikationssystem	Hohe Temperatur: schnellere Aushärtung Niedrige Temperatur: verzögerte Aushärtung
Relative Luftfeuchte	< 40 %: Verlängerung der Verarbeitungszeit um 1–3 min
Offene Lagerung des Pulvers	Wasseraufnahme und damit eine Veränderung der Misch- und Aushärteeigenschaften möglich
Pulver-Flüssigkeits-Verhältnis	Bei Nichteinhaltung der Herstellervorgaben Veränderung der mechanischen und Verarbeitungseigenschaften möglich
Zumischen von Wirkstoffen	Inhomogener Teig und Veränderung der Verarbeitungseigenschaften möglich
Handschuhe	Unterschiede bei der Bestimmung der Klebephase
Art der Anmischvariante (offenes Gefäß/Vakuumkartusche)	Unterschiede bei der Benetzung der Polymere mit Monomer und daraus resultierende Veränderung der Klebephase
Frequenz und Parameter der Durchmischung	Bei Nichteinhaltung der Herstellervorgaben inhomogener Teig und Veränderung der Verarbeitungseigenschaften möglich
Sterilisationsart (Gas‑/Strahlensterilisation)	Gassterilisation: geringer Einfluss auf die Verarbeitungseigenschaften Strahlensterilisation: Spaltung der Polymerketten und Verringerung des Molekulargewichts, dadurch größere Veränderungen der Verarbeitungseigenschaften

*ISO* Internationale Organisation für Normung

### Temperatur

Den größten Einfluss auf die Verarbeitungseigenschaften der Knochenzemente haben Temperatur und Feuchte. Dabei muss allerdings zwischen den Umgebungsbedingungen und der Temperatur der gelagerten oder bereitgestellten Knochenzement- und Anmischkomponenten unterschieden werden [31]. Der Einfluss eines zu kalten oder zu warmen Materials hat dabei einen wesentlich größeren Einfluss als eine kurzfristige Lagerung beziehungsweise Anwendung unter Umgebungsbedingungen. Grundsätzlich führen kühlere Umgebungen oder Komponenten zu einer langsameren Polymerisation und einem verzögerten Viskositätsanstieg, während höhere Temperaturen den gegenteiligen Effekt haben und den Viskositätsanstieg beschleunigen [21]. Daher sollte beim Umgang mit PMMA-Knochenzementen auch die Bereitstellungszeit im temperierten Operationssaal mit beachtet werden. Wird eine Zementpackung beispielsweise aus einem Lagerbereich mit einer stark abweichenden Temperatur (z. B. Kühllager) erst kurz vor dem Einsatz in den Operationssaal gebracht, haben die Komponenten Pulver und Flüssigkeit nicht genügend Zeit, um sich an die Umgebungsbedingungen anzupassen. Daher ist es empfehlenswert das Material vor der Anwendung 1–2 Stunden im Operationssaal zu konditionieren [18]. Damit ist sichergestellt, dass das Anmisch- und Verarbeitungsverhalten den Herstellerangaben entspricht.

### Vorkühlung der Zementkomponenten

Werden die Zementkomponenten eines hochviskosen Knochenzementes bei niedrigeren Temperatururen vorgekühlt, führt dies zu einer deutlich verringerten Anfangsviskosität sowie zu einer Verlangsamung der Polymerisation und damit auch des Aushärteverhaltens. Die Kühlung der Zementkomponenten wird in erster Linie vorgenommen, um in Vakuumanmischsystemen hochviskosen Zement bequem und leicht zu Mischen. Zudem wurde vermutet, man könne durch diesen Vorgang die Aushärtetemperatur reduzieren. Dies ist aber nicht der Fall, die maximale Temperatur tritt nur zeitlich später auf. Um den bestmöglichen Einfluss durch das gekühlte Zementmaterial zu erzielen, empfiehlt es sich, auch das verwendete Anmischgefäß oder Kartuschensystem mit vorzukühlen. Die Komponenten sollten erst kurz vor der Anwendung dem Kühlschrank entnommen werden, da die Komponenten die Umgebungstemperaturen rasch annehmen, besonders die Monomerflüssigkeit [19]. Die Kühlung der Zementkomponenten und der Einsatz von Vakuummischsystem führt zudem zu einer deutlichen Reduzierung der Porosität, was wiederum einen positiven Einfluss auf die mechanischen, insbesondere die mechanischen Ermüdungseigenschaften [16] und damit auch die Standzeit der Prothese hat [9, 29, 35]. Von einer Vorkühlung niedrigviskoser Zemente ist abzuraten, weil eine Anmischung unter Vakuum das Monomer zum Sieden bringen kann und Monomerblasen als Poren im ausgehärteten Zement zurückbleiben [18].

### Feuchte

Die relative Luftfeuchte hat im Vergleich zur Temperatur zwar einen wesentlich geringeren Einfluss, sollte aber insbesondere im Rahmen der Lagerung, Vorbereitung und Bereitstellung des Pulvers beachtet werden. Wird das Pulver bei einer Luftfeuchtigkeit von weniger als 40 % offen oder im gasdurchlässigen Primärbeutel gelagert, kann sich die Verarbeitungszeit um 1–3 min verlängern [18]. Daher sollte das Zementpulver erst kurz vor seinem Einsatz dem Feuchteschutz (in der Regel ein Aluminiumbeutel) entnommen und verarbeitet werden [18]. Bei antibiotikahaltigen Zementen könnte eine Lagerung bei einer hohen Luftfeuchte leichter zu einer Verklumpung von hydrophilen Wirkstoffen im Polymerpulver kommen.

### Pulver- und Flüssigkeitsverhältnis

Das vorgegebene Pulver-Flüssigkeits-Verhältnis der Knochenzemente – zumeist 2:1 – (z. B. 40 g Polymerpulver und 20 ml Monomerflüssigkeit) sollte nicht eigenmächtig verändert werden. Alle Qualitätsangaben zum jeweiligen PMMA-Knochenzement sind auf das vom Hersteller festgelegte Pulver-Flüssigkeits-Verhältnis abgestimmt. Abweichungen können erhebliche Veränderungen am Produkt hervorrufen. Die ISO 5833 [15] erlaubt lediglich herstellbedingte Schwankungen von ±5 % der deklarierten Werte [24]. Wird die Flüssigkeitsmenge deutlich erhöht (z. B. zwei 20-ml-Ampullen auf 40 g Pulver anstatt der vorgeschriebenen einen Ampulle) wird zwar die Viskosität gesenkt, die mechanischen Eigenschaften und Verarbeitungseigenschaften jedoch signifikant verändert [24]. Zudem erhöht sich die Aushärtetemperatur und der Restmonomergehalt deutlich. Wird mehr Polymerpulver verwendet, stimmen die Verarbeitungseigenschaften nicht mehr und die Homogenität des Teiges ist zweifelhaft [18].

### Zumischen von Antibiotika

Bei der manuellen Beimischung von Wirkstoffen können sich je nach Art und Menge die Viskositäts- und Verarbeitungseigenschaften signifikant ändern. Die perioperative Zumischung ist stets mit der Frage einer sicheren Homogenität verbunden. Zudem werden Antibiotika nicht in die Polymerketten eingebaut und schwächen die mechanische Festigkeit der Zementmatrix nachhaltig [12]. Die Beimischung von mehr als 10 % (z. B. 4 g Antibiotikum auf 40 g Polymerpulver) wird allgemein als kritisch angesehen [11]. Von der Beimischung flüssiger Antibiotika wird abgeraten, da diese einen signifikanten Einfluss auf nahezu alle Zementeigenschaften und insbesondere auf dessen mechanische Stabilität haben [11, 14, 25].

### Handschuhe

Auch die Wahl der Handschuhe kann für die Bestimmung der Verarbeitungseigenschaften und insbesondere der Ermittlung der Klebephase entscheidend sein [13]. Die Oberflächenbeschaffenheit der eingesetzten Handschuhe sowie mögliche Beschichtungen können beim Kontakt mit dem PMMA-Zement zu unerwünschten Reaktionen führen, da das Material je nach Handschuh mehr oder weniger stark haftet.

## Fazit für die Praxis

- Polymethacrylsäuremethylester(PMMA)-Knochenzemente werden als hochviskos (HV), mittelviskos (MV) und niedrigviskos (LV) im Markt angeboten.
- Die Teigviskosität wird durch die Zusammensetzung des Polymerpulvers beeinflusst.
- Die Polymerisation durchläuft vier Phasen: I Anmischphase (Vereinigen der Komponenten), II Klebephase (Wartephase bis zum Ende der Abbindezeit), III Verarbeitungs- und Applikationsphase (Zement kann eingesetzt werden), IV Aushärtephase (vollständige Härtung des Materials).
- Polymerisationen sind extrem temperaturabhängig: je höher, desto schneller, je niedriger, desto langsamer.
- Die ISO 5833 beschreibt für die Verarbeitungseigenschaften von PMMA-Knochenzementen die Prüfung und Methoden der Klebfreiheit („doughing time“) und der Aushärtezeit („setting time“).
- Temperatur, Feuchte, Anmischungsbedingungen, Pulver-Flüssigkeits-Verhältnis und Zusammensetzung der Zementrezeptur beeinflussen die Verarbeitungseigenschaften.
- Antibiotikazumischungen können die Verarbeitungseigenschaften beeinflussen.

## Abkürzungen

ASTM: American Society for Testing and Materials
DBPO: Dibenzoylperoxid
DmpT: N,N-Dimethyl-p-toluidin
HV: Hochviskos
ISO: Internationale Organisation für Normung
LV: Niedrigviskos
MMA: Methacrylsäuremethylester
MV: Mittelviskos
PMMA: Polymethacrylsäuremethylester

## References

[1] ASTM F451-21 (2021), Standard Specification for Acrylic Bone Cement, American Society for Testing and Materials (ASTM)

[2] Barrett D, Douglas AD, Bohannon MJ (2018) Cementing best practices in total knee arthroplasty, 095274-180717 DSUS. https://jnjinstitute.com/sites/default/files/2019-06/095274-180717-Cementing-Best-Practices.pdf. Zugegriffen: 18. Juni 2023

[3] BG RCI (2023) Gefahrstoffinformationssystem für Chemikalien, Methylmethacrylat (MMA). https://www.gischem.de/suche/dokument.htm?client_session_Dokument=211. Zugegriffen: 5. Apr. 2023

[4] Breusch SJ, Kühn K‑D (2003) Knochenzemente auf Basis von Polymethylmethacrylat. Orthopäde 32(1):41–50 12557085 10.1007/s00132-002-0411-0

[5] Breusch SJ, Draenert Y, Draenert K (1998) Die anatomische Basis des zementierten Femurstieles. Eine Vergleichsstudie zum geraden und anatomischen Design. Z Orthop Ihre Grenzgeb 136(6):554–559 10036745 10.1055/s-2008-1045185

[6] Charnley J (1970) Acrylic cement in orthopaedic surgery. Livingstone, Edinburgh

[7] Deb S (Hrsg) (2008) Orthopaedic bone cements. Elsevier, Cambridge. ISBN 978-1-84569-376‑3.

[8] Debrunner HU (1976) Untersuchungen zur Porosität von Knochenzementen. Arch Orthop Unfallchir 86(3):261–278 1008727 10.1007/BF00418904

[9] Draenert K (1988) Zur Praxis der Zementverankerung – Farbatlas, 2. Aufl. Art and Science, München

[10] Eyerer P, Jin R (1986) Einfluss der Anrührbedingungen von PMMA-Knochenzementen auf deren Eigenschaften. Teil 2: Analyse des Anrührvorganges, Biomedizinische Technik. Biomed Eng 31(1–2):11–18 3955163

[11] Frommelt L (2004) Lokale Antibiotikatherapie. Weshalb lokale Antibiotika bei der Behandlung von Infektionen am Bewegungsapparat? In: Schnettler R, Steinau HU (Hrsg) Septische Knochenchirurgie. Thieme, Stuttgart, S 82–90 10.1055/b-0034-56755. ISBN 3‑13-116981‑8

[12] Hansen E, Kühn K‑D (2022) Essentials of cemented knee arthroplasty. Springer, Berlin, Heidelberg

[13] He S, Scott C, Luise M, Edwards B, Higham P (2003) Effect of choice of surgical gloves on doughtime measurements of acrylic bone cement. Biomaterials 24(2):235–237 12419623 10.1016/s0142-9612(02)00295-8

[14] Hetzmannseder SH, Yuhan C, Clemens K, Kühn K‑D (2021) Properties of orthopaedic cements biomechanically little affected by exceptional use of liquid antibiotics. Orthop Surg 13(7):2153–2162 34605610 10.1111/os.12911 PMC8528991

[15] ISO 5833:2002-05 (2002) Chirurgische Implantate – Knochenzemente auf Basis von Acrylharz, Internationale Organisation für Normung (ISO)

[16] ISO 16402:2008 (2008) Implants for surgery—Acrylic resin cement—Flexural fatigue testing of acrylic resin cements used in orthopaedics, Internationale Organisation für Normung (ISO)

[17] Kühn K‑D, Ege W, Gopp U (2005) Acrylic bone cements: composition and properties. Orthop Clin North Am 36(1):17–28 15542119 10.1016/j.ocl.2004.06.010

[18] Kühn K‑D (2014) PMMA cements—are we aware what we are using? Springer, Berlin, Heidelberg 10.1007/978-3-642-41536-4

[19] Kühn K‑D (2001) Handling properties of polymethymetacrylate bone cements. In: Walenkamp G, Murray DW (Hrsg) Springer, Berlin, Heidelberg, S 17–26. ISBN 3‑540-41677‑3

[20] Kühn K‑D, Lieb E, Berberich C (2016) PMMA bone cement: what is the role of local antibotics? Maîtrise orthopédique, Proceeding of N°243, commission paritaire 1218T86410, S 12–18 (1148 2362)

[21] Kühn K‑D (2001) Knochenzemente für die Endoprothetik. Ein Aktueller Vergleich der physikalischen und chemischen Eigenschaften handelsüblicher PMMA-Zemente. Springer, Heidelberg, Berlin. ISBN 3‑540-41182‑8.

[22] Kühn K‑D, Höntzsch D (2015) Augmentation mit PMMA-Zement. Unfallchirurg 118:737–748. 10.1007/s00113-015-0059-y 26315391 10.1007/s00113-015-0059-y

[23] Kühn K‑D, Ege W, Gopp U (2005) Acrylic bone cements: mechanical and physical properties. Orthop Clin North Am 36(1):29–39 15542120 10.1016/j.ocl.2004.06.011

[24] Kühn K‑D, Jahnke M (2002) Anwendung der HACCP-Risikoanalyse am Beispiel eines parenteralen Medizinproduktes. Pharm Ind 64(2):179–186

[25] Kühn K‑D, Renz N, Trampuz A (2017) Lokale Antibiotikatherapie. Unfallchirurg 120(7):561–572 28643095 10.1007/s00113-017-0372-8

[26] Kühn K‑D, Gopp U, Weder JA (2002) Verarbeitungsverhalten von PMMA-Knochenzementen. Kunststoffe 92:102–104

[27] Lee AJ, Ling RS, Vangala SS (1978) Some clinically relevant variables affecting the mechanical behaviour of bone cement. Arch Orthop Trauma Surg 92(1):1–18 727906 10.1007/BF00381635

[28] Lee C (2005) Properties of bone cements: the mechanical properties of PMMA bone cement. In: Breusch S, Malchau H, Well-Cemented Total T (Hrsg) Hip arthroplasty. Springer, Berlin, Heidelberg, S 60–66

[29] Lewis G (1999) Effect of mixing method and storage temperature of cement constituents on the fatigue and porosity of acrylic bone cement. J Biomed Mater Res 48(2):143–149 10331907 10.1002/(sici)1097-4636(1999)48:2<143::aid-jbm8>3.0.co;2-8

[30] Malchau H, Herberts P, Ahnfelt L (1993) Prognosis of total hip replacement in Sweden. Follow-up of 92,675 operations performed 1978–1990. Acta Orthop Scand 64(5):497–506 8237312 10.3109/17453679308993679

[31] Meyer PR, Lautenschlager EP, Moore BK (1973) On the setting properties of acrylic bone cement. J Bone Joint Surg Am 55(1):149–156 4691652

[32] Sigmund IK, Gamper J, Antoni A, Pantotopoulos J, Funoics PT, Windhager R, Kühn K‑D (2018) Mixing technique of PMMA—bone cement determines the ideal insertion time point in cemented arthroplasty. J Surg. 10.29011/2575-9760.001153

[33] Soltész U, Schäfer R, Kühn K‑D (2004) Einfluss der Alterung auf das Ermüdungsverhalten von Knochenzementen. Biomaterialien 5:1

[34] Steiner O, Melanie K, Walter G, Peter T, Kühn K‑D (2021) Is benzoyl peroxide detectable under physiological conditions in orthopaedic cement? Int J Nano Biomater 10(1):34–49. 10.1504/IJNBM.2021.114692

[35] Wixson RL, Lautenschlager EP, Novak MA (1987) Vacuum mixing of acrylic bone cement. J Arthroplasty 2:141–149. 10.1016/s0883-5403(87)80021-9 3612140 10.1016/s0883-5403(87)80021-9

